# Herpes Simplex Virus-2 Hepatitis: A Case Report and Review of the Literature

**DOI:** 10.1155/2020/8613840

**Published:** 2020-02-20

**Authors:** Amna Ahmed, Alejandro Granillo, Ethan Burns, Kerri Glassner, Nishath Naseem, Christopher Force, Suzanne M. Crumley, Ashley Drews

**Affiliations:** ^1^Houston Methodist Hospital, Department of Medicine, 6550 Fannin Street, Houston, TX 77030, USA; ^2^Houston Methodist Hospital, Department of Gastroenterology and Hepatology, Houston, TX 77030, USA; ^3^Houston Methodist Hospital, Department of Pathology and Genomic Medicine, Houston, TX 77030, USA; ^4^Houston Methodist Hospital, Department of Infectious Diseases, Houston, TX 77030, USA

## Abstract

Herpes simplex virus (HSV) is a rare cause of hepatitis in pregnancy and the chronically immunosuppressed, with a high propensity to progress to acute liver failure (ALF) and death. Patients typically present with a nonspecific clinical picture that often delays diagnosis and treatment, contributing to the high mortality rate. We present a case of a young female on chronic prednisone and hydroxychloroquine for systemic lupus erythematosus (SLE) who was diagnosed with HSV-2 hepatitis after presenting with right-sided chest and abdominal discomfort. Despite early clinical deterioration, prompt initiation of therapy with intravenous acyclovir and methylprednisolone led to rapid improvement.

## 1. Introduction

HSV is the second most prevalent sexually transmitted infection (21.9%), posing a serious public health concern [1]. Symptomatic HSV can present with painful mucocutaneous vesicular lesions, and when left untreated can lead to complications including preterm labor, fatal infections in newborns, aseptic meningitis, transverse myelitis, and retinal necrosis [2]. An uncommon but highly fatal sequelae of HSV that primarily involves the immunosuppressed and pregnant population is hepatitis. The following is a case of an immunocompromised patient presenting with pleuritic chest pain who was ultimately diagnosed with HSV hepatitis.

## 2. Case Presentation

The patient is a 30-year-old African American female with a medical history suggestive of recent genital herpes and SLE on chronic maintenance therapy with hydroxychloroquine 200 mg twice daily and prednisone 5 mg daily who presented with two days of right-sided pleuritic chest pain, dyspnea, and a nonproductive cough. She also noted progressive right upper-quadrant abdominal pain, nausea, and decreased appetite. She denied fevers, chills, weight loss, or rashes. In the 48 hours prior to admission, she had taken an estimated 6 grams of acetaminophen for pain control. She was on day two of oral metronidazole for treatment of a recently diagnosed *Trichomonas vaginalis* infection and also reported several painful vesicular lesions on the outer portion of her vulva that had crusted over 1 week prior to admission. She is employed as a school teacher and had been near multiple sick children with upper respiratory symptoms; otherwise, she had no other sick contacts, recent travel, or animal or chemical exposures. She denied use of additional medications, supplements, vitamins, alcohol, tobacco, and illicit drugs.

On admission, the patient was afebrile and normotensive. Physical examination was notable for chest wall tenderness to palpation and right upper-quadrant abdominal tenderness without hepatomegaly, guarding, or rebound tenderness. She had no cervical, submandibular, axillary, or inguinal lymphadenopathy. Skin examination revealed no rashes and external genital exam did not reveal any vesicular lesions or discharge.

Initial laboratory values were significant for leukocytosis of 13.51 k/*μ*L with neutrophilic predominance (95.2%), lymphocytopenia (1.8%), hemoglobin of 12.8 g/dL, and platelets of 179 k/*μ*L. There were no electrolyte abnormalities, and kidney function was preserved. Her alanine aminotransferase (ALT) and aspartate aminotransferase (AST) were elevated (423 U/L and 339 U/L, respectively), and her alkaline phosphatase and bilirubin were normal. Her international normalized ratio (INR) was elevated (1.2). Drug screen was negative for illicit substances, and acetaminophen, salicylate, and blood alcohol levels were undetectable. An electrocardiogram and transthoracic echocardiography did not suggest pericarditis or endocarditis.

The patient's clinical status declined in the first 48 hours of admission. Her abdominal pain worsened and was now associated with nausea and fever of 38.5°C. She developed a rapidly rising bilirubin (total bilirubin 3.1 mg/dL), a dramatic increase in her liver transaminases (AST 2607 U/L and ALT 2364 U/L), worsening coagulopathy (INR of 1.4 mg/dL), and new onset thrombocytopenia of 107,000 k/*μ*L. Abdominal ultrasonography showed normal liver parenchyma without biliary ductal dilatation; Doppler ultrasonography showed patent portal and hepatic vasculature. Similarly, magnetic resonance cholangiopancreatography (MRCP) showed a normal liver and no biliary system abnormalities that would explain the patient's clinical and laboratory aberrancies. Complete infectious workup including blood and urine cultures, respiratory viral panel, hepatitis panel, Human Immunodeficiency Virus (HIV) 4^th^ generation assay, HIV polymerase chain reaction (PCR), chlamydia, and gonorrhea nucleic acid amplification urine assays were negative. Serum samples for viral PCR studies including *Epstein-Barr Virus* (EBV), herpes simplex virus-1 and herpes simplex virus-2, varicella zoster virus (VZV), and cytomegalovirus (CMV) were obtained.

**Figure 1:**
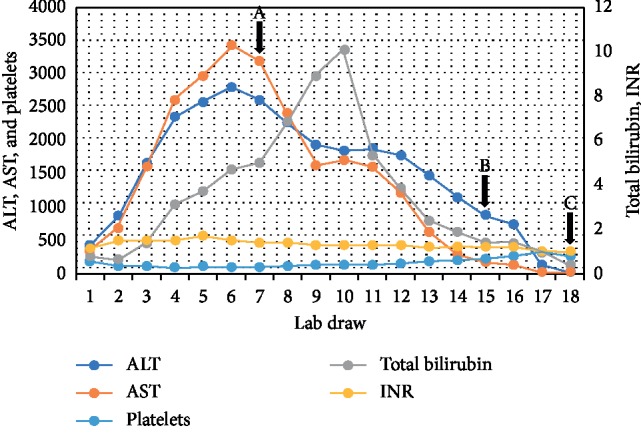
Trends of liver function tests (AST, ALT, and total bilirubin) and liver synthetic function (platelets and INR) over the course of this patient's hospitalization and follow-up visit. A. Initiation of intravenous acyclovir. B. Initiation of oral valacyclovir. C. One-month follow-up visit showing normalization of liver enzymes, platelets, and INR. AST: aspartate transaminase; ALT: alanine transaminase.

**Figure 2:**
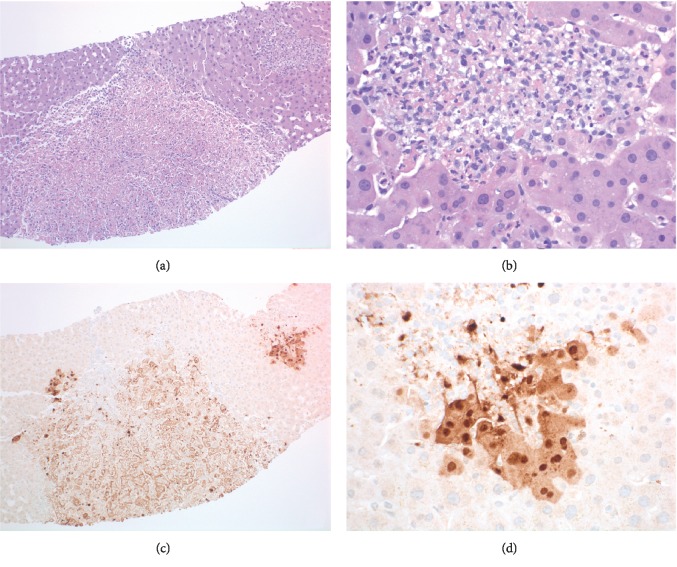
Transjugular core needle biopsies of the liver showed patchy involvement by well-demarcated areas of necrosis with associated inflammatory cells ((a) H&E stained slide, 100x magnification). The intervening liver parenchyma was unremarkable. A higher power view shows that the hepatocytes at the periphery of the areas of necrosis have glassy nuclear chromatin with margination and occasional multinucleation ((b) H&E stained slide, 400x magnification). Immunoperoxidase stains for herpes simplex virus (HSV-1/2) were performed and showed positive nuclear and cytoplasmic staining, characteristic of HSV-related hepatitis ((c) and (d) 100x and 400x magnification).

In the 72 hours following admission, the patient had further clinical deterioration with worsening fevers (39.2°C), lethargy, somnolence, severe abdominal pain, and nausea. Her AST and ALT peaked at 3428 U/L and 2597 U/L, respectively; coagulopathy continued to worsen (INR 1.7) and platelets continued to decrease (94 k/*μ*L) (Figure 1). Given clinical and laboratory deterioration, a transjugular liver biopsy was performed. Prior to finalized pathology results, serum HSV-2 PCR returned positive (viral load of 56,000,000 copies/mL). The patient was promptly initiated on intravenous acyclovir 15 mg/kg every 8 hours and methylprednisolone 40 mg every 8 hours. Liver histology showed patchy areas of necrosis involving 15% of sampled liver parenchyma (Figure 2, panels (a) and (b)) and HSV-1/2 was positive by immunostain, confirming the diagnosis of HSV hepatitis (Figure 2, panels (c) and (d)). The patient had marked clinical and laboratory improvement in the first 24 hours following acyclovir and methylprednisolone initiation. After 6 days of parenteral acyclovir, she was transitioned to high dose oral valacyclovir and her intravenous steroids were transitioned to oral prednisone. After 5 days of therapy, her repeat HSV viral load decreased to 2000000 copies/mL. At her 1-month follow-up clinic visit, liver function tests, INR, and platelets had normalized (Figure 1).

## 3. Discussion

Hepatitis secondary to infection with HSV serotype 1 or 2 is a rare diagnosis that has a propensity to rapidly progress to fulminant liver failure [3], with mortality rates that approach 90% [4]. HSV hepatitis most commonly affects patients with impaired immunity and pregnant women in their third trimester, though literature reports have reported up to 25% of cases in immunocompetent individuals [3]. Juhl and colleagues suggest that although HSV viremia is possible, it seems to be limited to primary infections [5]. In their study, no HSV viremia was able to be detected in patients with recurrent herpes labialis [5]. The patient in our case reported genital lesions observed by her one week prior to hospital admission and otherwise reported no previous episodes of genital lesions. She was presumed to have primary HSV-2 infection.

Patients often present with nonspecific flu-like symptoms including fever, myalgias, and abdominal pain [6,7]. The characteristic mucocutaneous herpetic skin lesions are found only in 30–50% of cases [4]. Common laboratory findings included leukopenia, thrombocytopenia, and coagulopathy. Renal failure and disseminated intravascular coagulopathy are reported less often, and encephalopathy, if seen, is often a late manifestation [3]. Approximately 90% of patients with HSV hepatitis have a characteristic liver profile known as “anicteric hepatitis,” which refers to a 100–1000-fold increase in transaminases, AST greater than ALT, with a relatively normal or low bilirubin [4, 8–12].

Liver ultrasonography may show enlargement of the liver and decrease in parenchymal echogenicity consistent with inflammation, which is neither specific nor diagnostic [13]. Computerized tomography and magnetic resonance imaging of the abdomen in viral hepatitis is nonspecific and includes hepatomegaly and periportal edema [14]. The gold standard diagnostic modality is histopathologic confirmation from liver biopsy which shows hemorrhagic necrosis, inflammation, enlarged ground glass nuclei with marginalized chromatin, and HSV immunostaining [3]. If liver biopsy is not available or cannot be done timely, clinical markers such as aminotransferases >500 U/L, fever, coagulopathy, encephalopathy, leukopenia, thrombocytopenia, and acute renal failure coupled with a positive HSV PCR should prompt the initiation of antiviral treatment [3].

In general, most cases of HSV hepatitis are diagnosed at autopsy [7], stemming from the rarity of the disease and low clinical suspicion during workup. However, if recognized and treated expediently, the disease process can be reversed [15]. The treatment of choice remains to be high-dose intravenous acyclovir 5–10 mg/kg every 8 hours for 2–7 days or until clinical improvement is observed, followed by oral antiviral therapy to complete at least 10 days of therapy [16]. When selecting the appropriate oral antiviral agent, the bioavailability must be considered. The prodrug valacyclovir hydrochloride, the l-valyl ester of acyclovir, has an oral bioavailability ranging from 44.9% to 54.5%, which is greater than oral acyclovir (26.9%) [17, 18], and thus in this case was the preferred antiviral. Since there is no standardized treatment data for HSV hepatitis, our patient was treated with acyclovir 10–15 mg/kg every 8 hours for 6 days, and transitioned to high-dose oral valacyclovir 1000 mg every 8 hours for a total of 28 days. Follow-up testing after completion of therapy showed normalization of her liver enzymes, platelets, and INR.

Despite expeditious initiation of appropriate antiviral therapy, morbidity and mortality remain high. Two available literature reviews reported that, with acyclovir treatment, mortality decreases to 51–55%, whereas those who did not receive treatment had a mortality rate ranging from 80 to 88% [7, 19]. This mortality benefit was obtained primarily if treatment with IV acyclovir was initiated within 4.2 days of symptom onset [7]. Associated findings that conferred a poorer prognosis included patients who were male, older, immunocompromised, and/or presenting with significant liver dysfunction [7]. In general, providers should consider initiating empiric acyclovir in patients with acute or impending liver failure without known etiology, especially if the patient is immunosuppressed or has a known history of mucocutaneous lesions consistent with HSV.

Superior survival has been demonstrated in patients with ALF stemming from viral hepatitis, although there is no differentiation made amongst the different viral etiologies [20]. The benefits of corticosteroid use in ALF remain a controversial topic and may further impact the immunocompromised status of the majority of the HSV hepatitis populace. However, in the setting of ALF or suspicion of impending ALF with rapid hepatic deterioration from HSV hepatitis, stress-dose steroids may act as an adjunct treatment modality to reduce morbidity and mortality and improve prognosis, as seen in the present case. Steroids are not often utilized in ALF secondary to viral hepatitis in the SLE population, and whether they confer a true benefit on overall survival remains uncertain. For example, Kluger et al. presented a case of HSV hepatitis in which a patient on chronic immunosuppressive therapy with steroids for SLE was managed with acyclovir in the absence of stress-dose steroids and recovered well [21]. The potential positive impact of stress-dose corticosteroid use in our patient should be corroborated by future studies.

## 4. Conclusion

HSV hepatitis is a rare and rapidly fatal manifestation of disseminated HSV that has been reported in both immunocompromised and immunocompetent individuals. A thorough history and physical examination including prior orogenital lesions, sexual activity or current pregnancy, and use of immunosuppressants is an important first step for evaluation. Laboratory workup suggestive of acute liver failure in a patient with the above risk factors should prompt the clinician to obtain serum viral PCRs including HSV. Although the gold standard for diagnosing HSV hepatitis is with liver biopsy, a positive HSV PCR in the setting of impending acute liver failure should prompt the initiation of acyclovir. If any degree of suspicion exists, clinicians should not wait for return of results prior to initiating empiric antiviral therapy. Stress-dose intravenous steroids should also be considered due to the potential mortality benefit, although further studies should be conducted to corroborate this. Once the patient's clinical picture has improved, clinicians should consider transitioning from IV acyclovir to oral valacyclovir given its superior bioavailability.

## Abbreviations

HSV: Herpes simplex virus
ALF: Acute liver failure
SLE: Systemic lupus erythematosus
